# Effectiveness of hygienic-dietary recommendations as enhancers of antidepressant treatment in patients with Depression: Study protocol of a randomized controlled trial

**DOI:** 10.1186/1471-2458-10-404

**Published:** 2010-07-09

**Authors:** Mauro Garcia-Toro, Olga Ibarra, Margalida Gili, Joan Salva, Saray Monzón, Margalida Vives, Maria J Serrano, Javier Garcia-Campayo, Miquel Roca

**Affiliations:** 1 Institut Universitari d'Investigació en Ciències de la Salut (IUNICS). Universitat de les Illes Balears (UIB). Red de Investigación en Actividades Preventivas y Promoción de la Salud (redIAPP). Spain; 2 Department of Psychiatry. Hospital Son Llatzer. Palma de Mallorca. Spain; 3 Department of Psychiatry. Hospital Son Dureta. Palma de Mallorca. Spain; 4 Department of Psychiatry. Hospital Joan March. Palma de Mallorca. Spain; 5 Department of Psychiatry, Hospital Miguel Servet, Universidad de Zaragoza, Zaragoza, Red de Investigación en Actividades Preventivas y Promoción de la Salud (redIAPP). Spain

## Abstract

**Background:**

In recent years some studies have been published supporting the efficacy of light exposure, physical activity, sleep control and a Mediterranean diet pattern on the improvement or prevention of Depression. However, to our knowledge, there have been no studies using all these measures together as an adjuvant antidepressant strategy.

**Methods:**

Multicenter, randomized, controlled, two arm-parallel, clinical trial. Eighty depressed patients undergoing standard antidepressant treatment will be advised to follow four additional hygienic-dietary recommendations about exercise, diet, sunlight exposure and sleep. Outcome measures will be assessed before and after the 6 month intervention period.

**Discussion:**

We expect the patients in the active recommendations group to experience a greater improvement in their depressive symptoms. If so, this would be a great support for doctors who might systematically recommend these simple and costless measures, especially in primary care.

**Trial Registration:**

ISRCTN59506583

## Background

Depression has become a serious public health burden, with high prevalence and morbidity rates [1,2]. The problem of depression, however, is not only one of health, but rather of poor work performance, absenteeism, medical visits, hospitalizations, and other expenses generated by unresolved depressive symptoms [3].

It is estimated that more than one in five patients do not respond to the first choice treatment and remain depressed more than two years later [4]. Furthermore, only half of those responding to antidepressants achieved complete remission [5]. It seems reasonable, therefore, to use any adjuvant therapeutic resource that can add some antidepressant efficacy without compromising safety and tolerability of standard treatments. In fact, some simple interventions based on hygienic and dietary habit changes, which could be considered almost costless and devoid of side effects, have been proposed. These potentially anti-depressant measures are summarized below.

### Exercise

Studies on the influence of exercise on affective disorders are methodologically difficult, but it is usually found to have a positive influence [6]. Furthermore, not only intense exercise, but regular moderate physical activity would be sufficient to exert positive antidepressant effects [7,8]. Some Major Depression Treatment Guidelines have even proposed exercise as a possible initial treatment in mild Depression [9].

### Diet

In developed societies there are several lifestyle factors, including food, which are receiving increased attention as a key factor in maintaining health. Unfortunately, the Mediterranean diet pattern, rich in vegetables and fish, is being replaced in many countries with a less heart-healthy diet: saturated fats, refined sugars and precooked products [10,11]. This unhealthy diet can increase the risk of an imbalance of omega polyunsaturated fatty acids and can lead as well to vitamin and mineral deficiencies. All of these factors can induce to a higher risk of Depression [12]. This would be consistent with the results of recent studies in which following a Mediterranean diet or adding supplements of vitamins or omega acids can have a positive effect on the prevention and treatment of Depression [11-14].

### Sunlight Exposure

The therapeutic potential of phototherapy has been discussed in last years, especially in a new subtype of psychiatric disorder: Seasonal Affective Disorder [15]. Some reviews support the use of light both in seasonal and non-seasonal Depression [16,17]. Exposure to sunlight has also been recommended as a "natural" light treatment [18].

### Sleep control

Several lines of research have established the relationship between sleep and mental well-being [19]. Any disturbance of a normal sleep pattern is associated with a clear functional limitation and a reduced quality of life. Sleep disturbances are core symptoms of Depression [20]. Some behavioral techniques have been proposed as useful to improving the quality of sleep of depressive patients, such as sleep scheduling and sleep restriction, reducing time in bed, avoiding daytime naps and stimulus control [21].

This paper aims to describe the design of a study to test the effectiveness of four hygienic-dietary recommendations as adjunctive treatment in Depression.

These four measures are: to engage in moderate physical activity for at least one hour a day, to be exposed to sunlight for at least two hours daily, to have a good night's sleep hygiene and to follow a Mediterranean diet pattern.

## Methods

### Study population

Eighty patients will be recruited, aged 18 or more, suffering from a Depression episode (Major Depressive Disorder, Dysthymic Disorder or Bipolar Disorder type I or II, depressed phase, according to DSM-IV) [22].

### Objectives

The main objective of the study is to test the potential benefit of giving a written series of structured hygienic-dietary recommendations to depressed patients. We will compare this group's response to the four specific recommended measures with a control group in which four general and nonspecific recommendations will be proposed. The study will last six months. Secondary goals will be to evaluate which of the various sociodemographic and clinical characteristics influence the effectiveness of the aforementioned recommendations.

### Study Design

This is a multicenter study, which includes the Son Dureta, Son Llàtzer and Joan March Hospitals, located in Palma de Mallorca. Eighty outpatients with a depressive episode will be randomly assigned either to the active treatment group or control group. All participants will continue standard antidepressant drug treatment controlled by their reference team.

### Inclusion Criteria

1. Male and female patients over 18 years.

2. Patients experiencing a depressive episode according to DSM-IV diagnostic criteria Major Depressive Disorder, Dysthymic Disorder or Bipolar Disorder.

3. Patients receiving antidepressant treatment.

5. Patients who have the ability to communicate in Spanish and give informed written consent.

6. Women of childbearing age practicing safe contraceptive methods.

### Exclusion Criteria

1. Patients suffering from another severe disease that affects the CNS (cerebral organic pathology) or who have suffered any serious head injury.

2. Other severe psychiatric illnesses (history of schizophrenia or other psychotic disorders, substance dependence or abuse and eating disorders).

3. Presence of a severe, uncontrolled, or potentially interfering medical condition.

4. Presence of delusions or hallucinations at the time of the study.

5. Significant risk of suicide. 6. Pregnancy or lactation.

### Diagnostic Tools and Measurements

*1. DSM-IV criteria for Major Depressive Disorder*. The 4th edition of "Diagnostic and Statistical Manual of Mental Disorders" is a widely use comprehensive classification of officially recognized psychiatric disorders [22]

2. *The Mini International Neuropsychiatric Interview (MINI) *is a short diagnostic structured interview designed to generate 17 DSM-IV or ICD10 axis I diagnosis [23].

3. *Sociodemographic and clinical questionnaire *designed specifically for this study. Relevant sociodemographic information regarding gender, date of birth and age, marital status, education level and occupational status, as well as clinical data regarding age of disorder onset, number of previous episodes and current pharmacological treatment (type of agent, mean dose and time of administration) will be collected.

4. *The Beck Depression Inventory (BDI) *is a test presented in multiple choice format which purports to measure presence and degree of depression. Each of the 21-items of the BDI attempts to assess a specific symptom or attitude which appear(s) to be specific to depressed patients, and which are consistent with descriptions of the depression contained in the psychiatric literature [24].

5. The *SF-36 *is a general measure that is intended to capture quality of life as well as whether an individual is healthy or not [25]. The SF-36 is made up of 8 scales. These cover the ability to function and complete everyday activities, including physical activities and social activities. The scales also capture well-being, such as energy or fatigue and mental health.

6. *17-item Hamilton Depression Rating Scale (HAMD)*. This is the most commonly used severity scale in clinical practice and research with mood disorders [26].

7. *The Global Clinical Impression rating scales (GCI) *measures symptom severity, treatment response and efficacy of treatments in patients with mental disorders [27].

### Trial Procedures: Consent, Baseline Assessments, Blinding and Randomization

Collaborating researchers of the three participating institutions will identify all those potential patients who might meet the required criteria. During the recruitment visit patients will be informed of every detail of the study, answering all questions and concerns that they might have regarding their participation. Signed informed consents will then be collected (Table 1). Researchers will confirm that patients meet the criteria required for entry into the study using the Structured Interview MINI [23]. The following two questionnaires will later be administered: Beck Depression Inventory of 21 items (BDI-21) [24] and Questionnaire on Health Status (SF- 36) [25]. Finally, an appointment will be arranged within a week. During this baseline interview, a blind assessor will quantify the affective symptomatology through the Hamilton 17-item scale (HAMD-17) [26] and Clinical Global Impression scale (CGI). Subjects will then be checked to ensure that they do not show organic pathology which might induce symptoms of depression (EKG, BP, HR, BMI). The following tests will also be administered: blood count, biochemistry, coagulation studies, thyroid function tests, serology, levels of vitamins (folic acid and vitamin B12), and urine test with toxic levels, in addition to a pregnancy test if appropriate.

**Table 1:** Study Schedule

	*WEEK 1/"TRIAGE"*	*WEEK O/BASELINE*	*WEEK 24*
Sociodemographic questionnaire		X	
			
Clinic questionnaire		X	X
			
MINI Interview	X		
			
Discussion of the study information leaflet	X		
			
Signed Consent Form	X		
			
Physical parameters			
• BP		X	X
• HR		X	X
• BMI		X	X
			
EKG		X	
			
Blood Test		X	X
			
Urine Test		X	X
			
Self Administered Test			
• BDI-21	X		X
• SF-36	X		X
			
Test administered by Blind Raters			
• HAMD-17		X	X
• GCI		X	X

Delivery of Hygienic-dietetic recommendations		X	

Patients will then be randomly assigned to one of two treatment arms according to sequence of entrance, using blocks of randomization. Patients assigned to the active treatment group will receive an envelope containing a sheet of paper with the four hygienic-dietary recommendations under consideration:

*1. Go to bed when sleepy and not before 11 o'clock at night. Use your bed and bedroom only for sleep and sex (do not read, watch TV or lie on the bed during the day). If you do not fall asleep after 15 or 20 minutes get up and start an activity until you feel sleepy enough to go back to bed. Get up early, never later than 9 a.m., no matter how well you have slept the night before. Do not lie down or take a nap during the day*.

*2. Walk at least 1 hour a day, at a good pace but without becoming short of breath or being unable to talk while walking. If you think you have a medical problem which makes walking difficult or uncomfortable consult your doctor. Use appropriate footwear for walking and have a shower or a bath afterwards*.

*3. Be exposed to sunlight at least 2 hours per day, taking precautions to avoid sunburn or sunstroke (sunscreen, hat, etc.)*.

*4. Try to eat a healthy and balanced diet. Eat at regular hours without snacking between meals. Avoid especially sweet or sugary drinks. Eat fish at least three times per week, plus fruit, cereals, nuts and vegetables daily*.

The control group will receive an identical envelope, but the advice will be to perform the pattern of eating, sleeping, exercise and exposure to light which they think will make them feel better:

*1. Sleep the hours that you feel your body needs*.

*2. Adapt the pace of daily physical activity to meet your needs best*.

*3. If exposed to sunlight take precautions to avoid sunburn or sunstroke (sunscreen, hat, etc.)*.

*4. Try to eat a healthy and balanced diet*.

After the 6th month patients will be visited and information will be sought about health resource utilization (hospitalizations, number of visits to the emergency department...) and work status. There will be a new collection of the same physical parameters, and blood and urine parameters will be studied. The following tests will again be collected: BDI-21, and SF-36. The blind rater will also repeat the questionnaires conducted at the beginning of the study: HAMD-17, and CGI.

### Outcome Measures

The primary variable of efficacy of the study will be the score of HAMD-17 obtained by the blind assessors before and after treatment. A full response is considered if the decline in scores on the HAMD-17 is greater than 50%, and a partial response if the reduction is between 25% and 50%. We will use BDI-21, filled out by the patient, and also the self-administered SF-36 and the GCI as secondary efficacy measures.

### Power calculation

To estimate the sample size, HAMD-17 final score was used. The value expected for control group is 12 (SD: 8) and the value expected for active treatment group is 8 (SD: 6). An alpha error of 5% is assumed and a beta error rate of 20% is accepted. The final sample is 34 for both samples. Expecting a 15-20% loss, the necessary sample size will be 40 patients in each group, constituting thus a total sample of 80 patients.

### Statistical Analyses

Obtained data will be analyzed with the Statistical Package for the Social Sciences (SPSS) version 17. There will be a descriptive analysis of baseline variables by comparing these variables between the groups. We will examine the outcome variables using the Student t-Fisher as mean comparison test. We will also use the analysis of variance (ANOVA) repeated means, the Mann-Whitney for comparisons involving ordinal variables and the Chi-square test for categorical variables. For the multivariate analysis linear and logistic regression models will be used.

### Ethics

The protocol has been approved by the Ethical Board of the Balearic Islands, Spain (n° IB 733/06 PI).

## Discussion

If we assume that the prevalence of depression is increasing in western countries, it is necessary to search for reasons which might explain it [1]. One reason could be the lifestyle of the population, which we know is changing in recent decades. A significant proportion of the population has reduced their physical activity while following a less healthy diet [8,11]. In addition, there is a tendency to sleep fewer hours and be less exposed to sunlight [18,19]. We know that these changes in lifestyle have an impact on brain physiology that may increase depression vulnerability [7,12]. There are also very suggestive evolutionary explanations in this regard. As a species we have evolved under the selective pressure of vigorous physical activity, essential for success in the harvesting, hunting and scavenging that allowed our ancestors to survive. This activity took place outdoors and conditioned our diet and our way of living. It is therefore possible that changes in lifestyle of the population in the developed world are contributing to increase depression vulnerability. This study is based on this hypothesis.

Sleep control, exercise, diet and sunlight exposure have been separately recommended by several research groups as treatment for patients with Depression [28-30]. However, there is very little research on the usefulness of combining these simple hygienic-dietetic measures. There is an open study that successfully combines three recommendations (exposure to sunlight, exercise and vitamin pills) in a sample of women without antidepressant treatment [28]. Other studies associate light therapy and exercise programs in subjects with sub-threshold depressive symptoms [29]. Finally, another study uses sleep deprivation and light therapy in combination with psychopharmacology in bipolar patients suffering depressive symptoms [30]. All of them have reported positive results. However, to our knowledge there has been no study that meets all of these recommendations combining sleep, exercise, diet and sunlight exposure. Our study meets this challenge in a "real word" design: heterogeneous samples of depressed patients, with few exclusion criteria and in normal clinical practice conditions.

These measures, especially exercise and dietary, may be useful to prevent or improve many other medical conditions besides Depression. For example, comprehensive lifestyle changes including a low-fat vegetarian diet and moderate exercise may be able to induce regression of even severe coronary atherosclerosis [31]. Asking the patient to follow the clearly written recommended measures will require very little time on the part of the doctor. There will be almost no cost to the patient or the health system. Adverse effects resulting from these recommendations should be not expected.

### Main Strengths and Limitations

This is one of the first studies to evaluate the effectiveness of a lifestyle change intervention on depressive patients as carried out by regular staff members from several centers. Due to the fact that this intervention will be done in daily practice conditions, conclusions could be generalized without reservation. This strengthens considerably the external validity of the trial. The main limitation might be found in possible evaluation bias. Blind raters are informed about the hypotheses of the study. This might make them aware to which group the patients have been assigned. An involuntary bias of their rating cannot then be discarded. However, BDI self administered test could help us to disregard this possibility.

In conclusion, it is our intention to study the possible benefits of lifestyle changes for patients suffering from Depression by using a simple combination of hygienic-dietary recommendations on a written piece of paper. If successful, this would be a great support for doctors who might systematically recommend these simple measures to patients with such a serious disorder. We believe this finding could be particularly important for primary care, where Depressive Disorders are very common, especially among patients who are more frequent attenders [2,32].

## Abbreviations

CNS: (Central Nervous System); BP: (Blood Pressure); HR: (Heart Rate); BMI: (Body Max Index); ECG: (Electrocardiogram)

## Competing interests

The authors declare that they have no competing interests.

## Authors' contributions

Olga Ibarra, Miguel Roca, Margalida Gili, Joan Salva, Saray Monzón, Margalida Vives, M° Jesús Serrano, Javier Garcia-Campayo, Mauro Garcia-Toro.

OI, MG, MR, MG, JG and JS were substantially involved in the conception of the study and participated in its design. OI, MG, MR, SM, MV and MS are participating in the coordination of the study and the acquisition of data. MG drafted the manuscript with the assistance of OI, MR, MG and JG. The other authors critically revised the text. The present publication has been approved by all involved.

## Pre-publication history

The pre-publication history for this paper can be accessed here:

http://www.biomedcentral.com/1471-2458/10/404/prepub
